# Identification of diurnal rhythmic blood markers in bronchial asthma

**DOI:** 10.1183/23120541.00161-2023

**Published:** 2023-07-03

**Authors:** Karolina Krakowiak, Robert J. Maidstone, Amlan Chakraborty, Alexandra C. Kendall, Anna Nicolaou, Polly Downton, Andreea-Daniela Cristian, Dave Singh, Andrew S.I. Loudon, David W. Ray, Hannah J. Durrington

**Affiliations:** 1 Faculty of Biology, Medicine and Health, University of Manchester, Manchester, UK; 2 NIHR Oxford Biomedical Research Centre, John Radcliffe Hospital, Oxford, UK; 3 Oxford Centre for Diabetes, Endocrinology and Metabolism, University of Oxford, Oxford, UK; 4 Medicines Evaluation Unit, University of Manchester, Manchester, UK; 5 Manchester University NHS Foundation Trust, Manchester, UK

## Abstract

**Rationale:**

Asthma is a rhythmic inflammatory disease of the airway, regulated by the circadian clock. “Spill-over” of airway inflammation into the systemic circulation occurs in asthma and is reflected in circulating immune cell repertoire. The objective of the present study was to determine how asthma impacts peripheral blood diurnal rhythmicity.

**Methods:**

10 healthy and 10 mild/moderate asthma participants were recruited to an overnight study. Blood was drawn every 6 h for 24 h.

**Main results:**

The molecular clock in blood cells in asthma is altered; *PER3* is significantly more rhythmic in asthma compared to healthy controls. Blood immune cell numbers oscillate throughout the day, in health and asthma. Peripheral blood mononucleocytes from asthma patients show significantly enhanced responses to immune stimulation and steroid suppression at 16:00 h, compared to at 04:00 h. Serum ceramides show complex changes in asthma: some losing and others gaining rhythmicity.

**Conclusions:**

This is the first report showing that asthma is associated with a gain in peripheral blood molecular clock rhythmicity. Whether the blood clock is responding to rhythmic signals received from the lung or driving rhythmic pathology within the lung itself is not clear. Dynamic changes occur in serum ceramides in asthma, probably reflecting systemic inflammatory action. The enhanced responses of asthma blood immune cells to glucocorticoid at 16:00 h may explain why steroid administration is more effective at this time.

## Tweetable abstract

**The peripheral blood molecular clock in asthma demonstrates a gain in rhythmicity and blood immune cells from patients with asthma show significantly enhanced responses to immune stimulation and steroid suppression in the afternoon compared to overnight** https://bit.ly/3UwyVoZ

## Introduction

Marked diurnal variation in symptoms, airway narrowing and airway inflammation, is a hallmark of asthma [1]. The effectiveness of anti-inflammatory treatments in asthma may be affected by biological timing (chronotherapy) [2, 3]. Daily fluctuation in airway narrowing in asthma is linked directly to the body's internal timing system, the circadian system [4]. Circadian rhythms are generated by a molecular clock expressed in virtually all cells. A central clock in the suprachiasmatic nucleus of the brain synchronises peripheral tissue clocks *via* neural and humoral mediators. The cellular circadian molecular clock consists of transcription factors, a positive arm (CLOCK and BMAL1 heterodimers) driving transcription of two inhibitory arms (PER/CRY and REVERBs/RORs), which feed back to inhibit either BMAL1/CLOCK heterodimer transactivation function, or BMAL1 transcription, respectively.

Daily oscillation in clock transcription factors leads to subsequent rhythms in transcription of downstream gene targets [5, 6]. The circadian clock powerfully regulates inflammation [6]. Using a murine model of allergic asthma, we have shown the importance of the molecular clock in determining airway hyperresponsiveness [7] through NR1D1. However, little is known about the molecular clock in human asthma. Two previous studies have shown reduced clock gene expression in bronchial epithelial cells and in peripheral blood mononucleocytes (PBMCs) from patients with asthma, compared to healthy individuals [8, 9]. In both these studies, serial samples at different circadian phases were not collected, therefore no inference could be made about clock gene expression over time, throughout the day.

Asthma is regarded as an inflammatory disease of the airway; however, it is now well-recognised that there is “spill-over” of inflammation outside of the lungs, into the systemic circulation [10]. Blood biomarkers, such as eosinophils and periostin, may be used to diagnose and manage asthma in the clinic [11]. Moreover, other chronic inflammatory conditions, such as rheumatoid arthritis, have been shown to impact other organs, such as the liver, affecting production of serum ceramides (potent lipid mediators of inflammation) [12, 13]. Serum ceramides play an important role in asthma and in determining disease severity [14–16] and are robust and convenient circulating circadian biomarkers [17].

Here we used serial sampling to determine for the first time how clock gene expression changes over 24 h in whole-blood samples collected from a cohort of individuals with mild/moderate asthma compared to healthy controls. Our previous data demonstrated significant diurnal variation in lung function, airway eosinophils, fractional expired nitric oxide, breath volatile organic compounds and serum cortisol levels in this cohort of individuals [1, 18, 19]. Blood has the advantage of being easily accessible for repeated sampling, without perturbing lung tissue, or the asthma disease processes in the lung. We identify changes in the operation of the circadian clock machinery in circulating immune cells and find that time of day affects immune cell responses *ex vivo*. These findings suggest that the reason anti-asthma drugs vary in efficacy by time of dosing (chronotherapy [2, 3]) may relate to the circadian phase of the immune cell targets.

## Methods

10 healthy and 10 individuals with mild atopic asthma were recruited overnight and written consent was obtained [1, 18, 19]. Clinical details of the individuals included in the study have been published previously [1, 18, 19].

### Quantitative PCR for clock gene expression

6-hourly blood samples were drawn into PAXgene Blood RNA Tubes (BD Biosciences; 762165). Under ethics approval (research ethics committee identifier 19/NM/0057), RNA was extracted (Stabilized Blood-to-CT Nucleic Acid Preparation Kit; Life Technologies; 4449079), converted to DNA (GoScript Reverse Transcriptase; Promega; A5001) and underwent subsequent quantitative PCR for clock gene expression (KAPA SYBR FAST SYBR Green Master Mix; Merck; KK4601). Custom SYBR-green primers (Sigma, MO, USA) and pre-designed primers (Qiagen, the Netherlands) were acquired. Gene expression was quantified using the comparative Ct method to housekeeping gene 18s and reported relative to healthy 10:00 h sample, as relative quantification.

### Lipidomic analysis

Ceramides, phosphorylated ceramides, sphingoid bases, phosphorylated sphingoid bases and sphingomyelins were extracted from serum (500 µL) using isopropanol:water:ethyl acetate (3:1:6; 4 mL per sample). Internal standards were added (50 pmol; Avanti Polar Lipids, Alabaster, AL, USA) and samples incubated on ice for 30 min. 3 mL of sample was dried, reconstituted in methanol with 0.1% formic acid, for analysis of ceramides, bases and phosphorylated species. The remaining 1 mL was extracted for sphingomyelins in 1:1:0.9 chloroform:methanol:water, the organic phase removed, dried, reconstituted in methanol (1 mL), alkaline hydrolysis performed and incubated for 2 h in darkness. The pH was neutralised, the sample dried and reconstituted in methanol with 0.1% formic acid. Samples were analysed using ultraperformance liquid chromatography with electrospray ionisation and tandem mass spectrometry (UPLC/ESI-MS/MS) (Waters, Wilmslow, UK) [20]. Analytes were identified by multiple reaction monitoring in the positive mode and relative quantitation performed using class-specific internal standards.

### Serum periostin

Serum periostin concentration was measured using ELISA (EHPOSTN; Thermo Fisher).

### Blood immune cells

Whole blood was analysed automatically for differential leukocyte counts.

### PBMC analysis

PBMCs were immediately isolated from blood drawn at 16:00 h and 04:00 h using Ficoll gradient, and incubated ±dexamethasone (100 nM; Sigma D4902) for 15 min before stimulation with lipopolysaccharide (LPS; 10 ng·mL^−1^; Sigma L4391) and Dynabeads (CD3/CD28 Human T-Activator (25 µL/1×10^6^ cells); Thermo Fisher 11131D) for 2 h. Cytokines were measured in conditioned media (Cytokine & Chemokine Convenience 34-Plex Human ProcartaPlex Panel 1A (EPXR340-12167-901; Thermo Fisher)).

### Statistical methods

Significant differences for each gene at a single time point was determined by Mann–Whitney U-test. Two-way ANOVA with Tukey correction was used to determine differences between cell counts, as well as paired t-test for individual differences between time points. Differences between treatment groups were determined using unpaired t-tests with Bonferroni–Dunn for multiple corrections for each cytokine.

Gaussian processes have been used previously to detect oscillations in biomedical research [12, 18, 21]. Here, a hierarchical Gaussian process was used to capture patterns over time in both healthy and asthma cohorts. Firstly, data were z-normalised on each gene, for each individual. A hierarchical Gaussian process was fitted; a Gaussian process was fitted to each individual/gene pair with an exponential covariance function and mean taking a value of a second Gaussian process, shared across individuals, with a periodic kernel (period=24). Empirical p-values were obtained by comparing the residual sum of squares of the fit to the fits on a null distribution of nonrhythmic data. This approach was used for analysis of ceramides.

## Results

### Whole-blood clock gene expression in asthma

**FIGURE 1:**
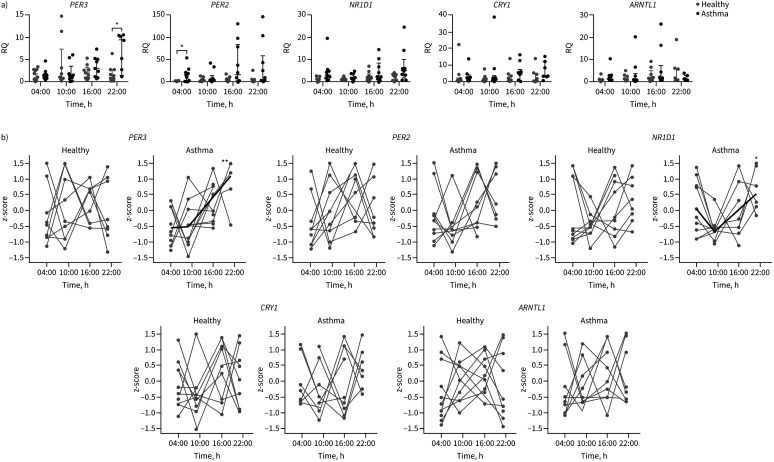
Clock gene expression in whole-blood samples drawn from 10 healthy and 10 asthma participants. a) Quantitative PCR data showing clock gene expression in whole blood drawn every 6 h relative to the housekeeping gene 18s and compared to healthy 10:00 h samples (relative quantification (RQ)). Data are presented as median (interquartile range). There is a significant increase in the expression of *PER3* at 22:00 h and *PER2* at 04:00 h in asthma compared to health (Mann–Whitney U-test with Bonferroni correction for multiple comparisons, p<0.05). b) PCR data were z-scored and hierarchical Gaussian processing applied to determine significantly rhythmic gene expression. Both *PER3* and *NR1D1* are rhythmic in asthma (p<0.01 and p=0.03, respectively), but not in health. After adjusting for false discovery rate (FDR), only *PER3* remained significantly rhythmic in asthma compared to health (*PER3* FDR=0.01, *NR1D1* FDR=0.06). *: p<0.05, **: p<0.01.

Clock gene expression was measured in whole blood collected 6-hourly from 10 mild/moderate atopic asthma patients and 10 healthy individuals (figure 1a). There was a significant increase in the expression of *PER3* at 22:00 h (p<0.05) and *PER2* at 04:00 h (p<0.05) in asthma compared to healthy samples (figure 1a). Overall, there was more variation in the expression of *PER2*, *PER3* and *NR1D1* at later time points (16:00 h and 22:00 h) compared to morning time points (04:00 h and 10:00 h) in asthma compared to health. The data were z-scored and using a hierarchical Gaussian processing method we determined which genes showed significant circadian rhythmicity. Both *PER3* and *NR1D1* were significantly rhythmic in those with asthma (p<0.01 and p<0.05, respectively), but not in healthy people (figure 1b and supplementary figure S1). After adjusting for false discovery rate (FDR), *PER3* remained significantly circadian rhythmic in asthma, compared to health (FDR=0.01) (figure 1b).

The *PER3* gene is associated with chronotype (morningness/eveningness) [22]. Genetic variation at *PER3* affects chronotype, and ease of waking in the morning. We found no difference in chronotype between healthy and asthma groups, nor in serum cortisol peaks [19], although the study was small and lacked power for this comparison.

### Rhythmic serum ceramides in asthma

**FIGURE 2:**
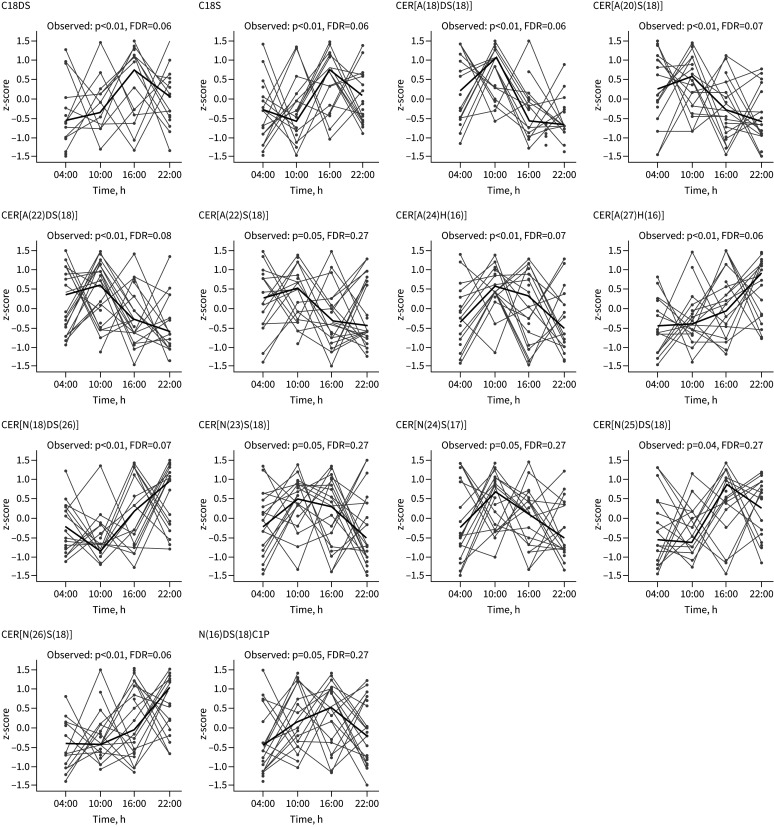
Gaussian processing analysis for rhythmic ceramides in serum from both healthy and asthma participants combined. Serum was separated from blood drawn 6-hourly from 10 healthy and 10 asthma participants. Lipidomic analysis of ceramides and phosphorylated species was performed through ultraperformance liquid chromatography with electrospray ionisation and tandem mass spectrometry of serum samples and the results analysed for rhythmicity using Gaussian processing. The figure shows rhythmic species identified when data from both healthy and asthma were analysed together (statistical significance is shown by p-value and corrected for false discovery rate (FDR)).

Period proteins (PERs) play a major role in lipid metabolism [23–25], specifically in regulating rhythmic expression of ceramide and ceramide-related genes [26]. Ceramides, the central metabolites of the sphingolipid pathway [14] are bioactive signalling molecules involved in regulation of several biological processes that may contribute to asthma [15, 27, 28]. Specific ceramide species may have potential to serve as biomarkers for specific asthma endotypes and disease severity [29]. We reported previously that serum ceramides are robust circadian biomarkers affected by chronic inflammation [12, 13]. Therefore, we performed targeted lipidomic analysis using UPLC/ESI-MS/MS of serum samples taken at 6-hourly intervals from both groups, to determine newly rhythmic lipid species in asthma. Perseus (version 1.6) determined mean lipid levels (with a permutation-based FDR). We identified 45 ceramides and three phosphorylated ceramides, 24 sphingomyelins, two sphingoid bases and two phosphorylated bases. Rhythmic ceramides were identified by combining results from healthy and asthma groups (n=20) and using a Gaussian process regression with a periodic kernel. In total, 14 ceramides were rhythmic; however, after correcting for FDR, none remained significantly rhythmic, although six out of 14 had FDR of 0.06, approaching the arbitrary significance threshold (figure 2). Next, we used Gaussian process regression to analyse the healthy and asthma groups separately. Four ceramides were rhythmic in both healthy and asthma groups; eight ceramides were only rhythmic in asthma; and four only rhythmic in health. Again, after correction for FDR, none remained significantly rhythmic, although CER[A(18)DS(18)] had a FDR of 0.08 in asthma (table 1 and supplementary figure S2).

**TABLE 1:** Gaussian processing to determine rhythmic serum ceramides in mild/moderate asthma (n=10) and in healthy (n=10) participants

	**Rhythmic in:**			
	**Healthy**		**Asthma**	
	**p-value**	**FDR**	**p-value**	**FDR**
**CER[A(24)H(16)]**	0.03	0.34	0.04	0.27
**CER[A(18)DS(18)]**	0.02	0.34	<0.01	0.08
**CER[A(27)H(16)]**	<0.01	0.3	0.02	0.19
**CER[N(26)S(18)]**	<0.01	0.3	0.02	0.19
**CER[A(20)S(18)]**	ns		<0.01	0.11
**CER[A(22)S(18)]**	ns		0.01	0.19
**CER[N(14)DS(18)]**	ns		0.05	0.27
**N(16)DS(18)C1P**	ns		0.03	0.23
**CER[N(18)S(18)]**	ns		0.02	0.19
**CER[N(25)DS(18)]**	ns		<0.01	0.15
**CER[N(18)DS(24)]**	ns		0.05	0.27
**CER[N(20)S(18)]**	ns		0.04	0.27
**CER[N(24)S(19)]**	0.02	0.24	ns	
**CER[N(23)S(18)]**	0.05	0.44	ns	
**CER[A(22)DS(18)]**	0.02	0.34	ns	
**CER[N(23)S(20)]**	0.04	0.41	ns	

Statistical significance shown by p-value and corrected for false discovery rate (FDR).

### Serum periostin

**FIGURE 3:**
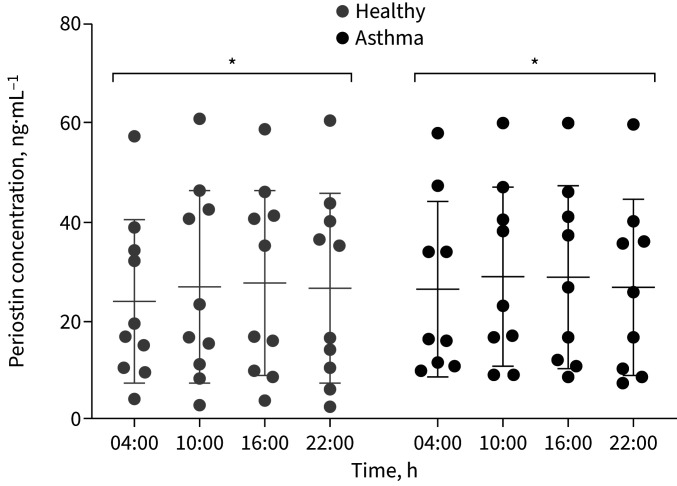
Serum periostin concentration from blood drawn 6-hourly from 10 healthy and 10 asthma participants. Serum was separated from blood drawn 6-hourly from 10 healthy and 10 asthma participants. Perisotin concentration was measured using ELISA. There were significant time-of-day differences in serum periostin levels across both healthy and asthma individuals (mean±sem, two-way ANOVA with Tukey correction). There were no significant differences between groups. *: p<0.05.

Serum periostin varied by time of day across both asthma and healthy groups, but there was no difference between the groups (p≤0.05, two-way ANOVA with Tukey correction) (figure 3).

### Blood immune cells in asthma

**FIGURE 4:**
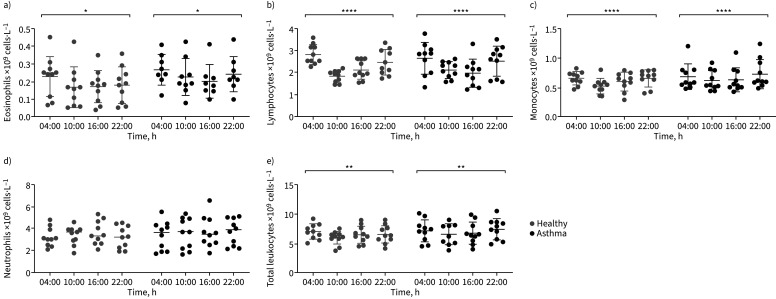
Blood immune cell number varies rhythmically in asthma and in health. There were significant time-of-day differences in blood a) eosinophil, b) lymphocyte, c) monocyte and e) total leukocyte numbers in both healthy and asthma individuals, but no difference between the groups (mean±sem, two-way ANOVA with Tukey correction). d) Neutrophil counts. *: p<0.05 for eosinophils in health and asthma; **: p<0.01 for total leukocytes in health and asthma; ****: p<0.0001 for both monocytes and lymphocytes in health and asthma.

**FIGURE 5:**
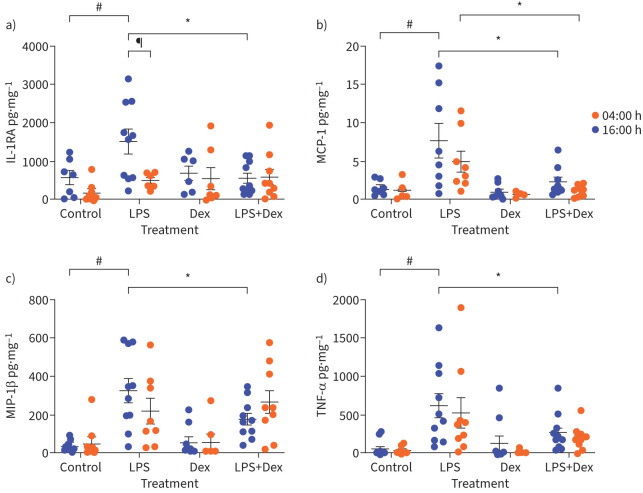
Peripheral blood mononuclear cells (PBMCs) from asthma subjects show different time-of-day responses to dexamethasone (Dex) suppression following lipopolysaccharide (LPS) stimulation. PBMCs drawn at 04:00 h and 16:00 h were isolated immediately using a Ficoll gradient, and incubated ±Dex (100 nM) for 15 min before stimulation with LPS (10 ng·mL^−1^) and CD3/CD28 T-cell Activator (25 µL/1×10^6^ cells) for 2 h. Cytokine concentration was measured in conditioned media. a) Interleukin-1 receptor antagonist (IL-1RA), b) monocyte chemoattractant protein **(**MCP)-1, c) macrophage inflammatory protein (MIP)-1β and d) tumour necrosis factor (TNF)-α increased in response to LPS in asthma (this was significant at 16:00 h, paired t-test, ^#^: p<0.05). IL-1RA showed a significant time-of-day difference following LPS stimulation (^¶^: p<0.05). IL-1RA, MCP-1, MIP-1β and TNF-α were significantly sensitive to Dex following LPS stimulation at 16:00 h; only MCP-1 reduced significantly at both 04:00 h and 16:00 h. *: p<0.05 unpaired t-tests with Bonferroni–Dunn for multiple corrections.

We determined *PER3* expression using RNA extracted from whole blood. Whole blood contains leukocytes, thrombocytes and erythrocytes; however, the majority of RNA (extracted for clock gene analysis) derives from leukocytes. We determined how blood leukocyte populations varied in number throughout the day in healthy and asthma groups (figure 4a–e). There were significant time-of-day differences in numbers of blood eosinophils, lymphocytes and monocytes in both healthy and asthma groups, peaking overnight and reaching a nadir in the afternoon, but no difference between the groups (two-way ANOVA with Tukey correction; p<0.05 for eosinophils in healthy and asthma, p<0.0001 for both monocytes and lymphocytes and p<0.01 for total leukocytes in healthy and asthma) (figure 4a–e). Neutrophils were not significantly rhythmic in either group (figure 4d). Since blood lymphocyte and monocyte number were the most rhythmic, we determined whether the response of these cells to an immune stimulus ±dexamethasone suppression was impacted by time of day. 16:00 h and 04:00 h are important clinical time points in asthma, corresponding to the peak (16:00 h) and nadir (04:00 h) of lung function variation [1, 4]. Furthermore, there is increasing evidence to suggest that steroids administered at 16:00 h rather than in the morning or evening might be more efficacious in treating asthma [2, 3]. PBMCs derived from a Ficoll gradient are largely lymphocytes and monocytes (and devoid of granulocytes, platelets and reticulocytes) [30]. We determined if there was a time-of-day difference in the response of PBMCs to combined LPS and CD3/CD28 stimulation in the presence or absence of dexamethasone suppression. Stimulating PBMCs with a combination of low-dose LPS and Human T-Activator CD3/CD28 allows us to replicate some of the complex immune pathways at play in asthma [31, 32]. PBMCs were extracted from blood collected from healthy and asthma individuals at 04:00 h and 16:00 h and stimulated with LPS (10 ng·mL^−1^) and a CD3/CD28 Human T-Activator (25 µL/1×10^6^ cells)±dexamethasone (100 nM) for 2 h. Cytokine concentration was measured in conditioned media. Only PBMCs from individuals with asthma showed significant responses to LPS/CD3/CD28 stimulation; no significant changes were observed in PBMCs from healthy individuals (figure 5a–d, supplementary figures S3 and S4). Among 14 analytes measured in conditioned media from individuals with asthma, nine significantly increased after stimulation with LPS and CD3/CD28 activation compared to untreated controls (supplementary figure S3a–e), and of these, four were suppressed with dexamethasone treatment (figure 5a–d). Interleukin (IL)-1α, IL-10, IL-17α and IL-6 increased significantly with LPS stimulation at both 16:00 h and 04:00 h (supplementary figure S3a–d), whereas IL-1 receptor antagonist (IL-1RA), monocyte chemoattractant protein (MCP)-1, macrophage inflammatory protein (MIP)-1β, tumour necrosis factor (TNF)-α and MIP-1α increased significantly at 16:00 h only (figure 5a–d and supplementary figure S3e). IL-1RA showed a significant time-of-day response to LPS stimulation, being increased at 16:00 h compared to 04:00 h (p<0.05). IL-1RA, MCP-1, MIP-1β and TNF-α showed suppression to dexamethasone; this was only significant in the asthma group (figure 5a–d). The LPS-mediated activation of IL-1RA, MIP-1β and TNF-α was attenuated significantly by dexamethasone at 16:00 h but not at 04:00 h (figure 5a, c and d), revealing a striking time-of-day dependence to both immune stimulation and glucocorticoid response. Only MCP-1 was inhibited by dexamethasone at both 04:00 h and 16:00 h in asthma (both p<0.05) (figure 5b).

There was a trend for increased cytokine concentrations after LPS stimulation of healthy PBMCs at both 16:00 h and 04:00 h, but no significant differences were found after dexamethasone suppression (supplementary figure S4).

## Discussion

By analysing sequential blood samples from individuals with mild/moderate asthma, we have shown for the first time that the molecular clock is altered in asthma compared to health. We have identified a group of rhythmic ceramides in the blood that are altered by disease state and shown that time of day is important in governing how PBMCs from patients with asthma respond to immune stimulation and steroid suppression, providing an insight into why chronotherapy with steroids may be important for asthma.

This is the first time that the peripheral clock in asthma has been shown to be significantly more rhythmic compared to healthy physiological rhythms. Ehlers *et al*. [8] showed a reduction in expression of *BMAL1*, *PER2* and *NR1D1* in bronchial brushings from mild/moderate asthma patients; however, only a single time point was evaluated. Similarly, Chen *et al*. [9] showed overall a dampening of clock gene expression in PBMCs from patients with bronchial asthma at a single, unspecified time point. Our data highlight the importance of serial sampling for cyclical clock gene expression and suggest that peripheral clocks in asthma are altered; whether clock genes in blood cells in asthma are driving changes in asthma or simply responding to inflammatory signals from the lungs remains to be elucidated. Here, we sampled every 6 h over a 24 h period. This was to minimise blood loss and inconvenience to participants. This sampling frequency reduces the power to detect circadian rhythms, but the comparison we sought was between the healthy volunteers and those with asthma, and previous analysis of this cohort had revealed rhythmic changes in lung function, sputum eosinophilia and breath biomarkers [1, 18, 19].

Analysing clock gene rhythms in whole blood has been reported previously [33]; as blood circulates throughout the body, gene expression analysis of whole-blood cells is potentially a novel tool to assess an individual's biological status. We found no difference in blood immune cell numbers between asthma and health in our study to account for the increased *PER3* expression found in asthma.

Previous studies have shown that *PER3* is important in the fine adjustment of the time-keeping system in humans, particularly in the pituitary and lung [34], and is also associated with lung function in children and adolescents [35].

Although we identified several serum ceramides as rhythmic, once adjusted for FDR these lost significance; however, many had low FDRs (≤0.08), approaching significance. We powered the study for circadian gene oscillation, and it may be that the study was underpowered for the exploratory ceramide analysis, and in particular the comparison of many ceramide metabolites. Despite this, it is interesting that many of the ceramides identified here as rhythmic feature an 18-carbon sphingoid base. These are the most abundant serum ceramides and it is possible that their concentrations made identification possible. However, two of the ceramides identified in the present study (CER[N(18)S(18)] and CER[N(20)S(18)]), were also identified by Reinke *et al.* [36] as being associated with increased asthma severity (“Ceramide(C18:0)” and “Ceramide(C20:0)”). Additionally, three species identified here (CER[A(18)DS(18)]) CER[N(26)S(18)] and CER[N(20)S(18)]) were identified by us as rhythmic in serum of patients with rheumatoid arthritis and/or in the liver of a mouse model of rheumatoid arthritis [12, 13]. The majority of circulating ceramides probably originate from the liver, so gains in rhythmicity in ceramide synthase enzymes expressed in the liver could contribute to the serum ceramide changes [12]. Downton *et al*. [13] revealed substantial metabolic changes occurring during inflammatory disease, leading to considerable mobilisation of lipids, particularly ceramides. This raises the possibility that serum ceramides may represent useful biomarkers for inflammatory diseases such as asthma and arthritis.

Serum periostin showed significant time-of-day variation in both asthma and healthy individuals; however, during the clinical working day (10:00 h and 16:00 h), serum periostin levels remained stable in asthma (and healthy people) and so would be unlikely to influence asthma management decisions, as reported previously [37].

Chronotherapy is the timing of drug administration to coincide with peak biological disease activity. Restricting dosing to a specific time of the day leads to greater drug effectiveness and reduces side-effects. In asthma, chronotherapy studies have shown that inhaled and systemic corticosteroids taken in the afternoon are as effective, if not more effective, than if taken in the morning or as divided doses during the day [2, 3, 38]. Here, for the first time, we have shown that PBMCs from patients with asthma demonstrate a time-of-day difference in their response to LPS with CD3/CD28 stimulation, as well as to dexamethasone suppression. Our results suggest that blood immune cells from patients with asthma appear more responsive to immune signals (both inflammatory and suppressive) during the active part of the day (afternoon) compared to during the inactive (rest) part of the day (night-time). Here we showed a peak in the number of circulating leukocytes occurring during the rest phase (night-time) in both healthy and asthma patients, as has been reported previously [39]. Leukocyte recruitment to tissue from the blood compartment is mediated by expression of chemokines and cell adhesion molecules which occurs during the active phase; this would fit with our results, where PBMCs from patients with asthma tended to be more responsive to LPS and CD3/CD28 stimulation and dexamethasone suppression at 16:00 h (during the active phase) [39]. Furthermore, mice exhibit an enhanced sensitivity to detect and respond to pathogens during the active phase owing to increased expression of components of the innate immune system [39]. Our results and those of others point to the active phase (afternoon) being a crucial time for the immune system and may present a window of opportunity for the most efficacious time to take asthma treatment.

A low-concentration LPS stimulation (10 ng·mL^−1^) in combination with CD3/CD28 activation was used in PBMC experiments, since LPS is ubiquitous in the environment and may influence the development of asthma [33]. Even at this low dose, LPS produces a fast and reproducible response after incubation with PBMCs for only 2 h, and since we were interested in time-of-day responses, this was an important factor in selecting an immune stimulus. Co-stimulation *via* CD28 is the most important pathway for the initial activation of naïve T-cells, a key cell type in asthma [32].

Our study has several limitations: all participants with asthma were taking moderate doses of inhaled corticosteroid [1], and although doses were omitted on the day of clinical measurements, it is possible that our results were affected by this. A larger, adequately powered, prospective study is required to appraise ceramide as an asthma biomarker and to determine whether chronotherapy has a role in asthma management. Future studies using RNA sequencing techniques may establish which pathways are driving the time-of-day responses of PBMCs to glucocorticoids.

In summary, we have shown the following for the first time: 1) the molecular clock is altered in asthma in the peripheral blood; 2) blood immune cells from patients with asthma show a time-of-day difference in how they respond to immune signals; and 3) serum ceramides become newly rhythmic in asthma, perhaps due to a change in liver metabolism.

## Supplementary material

10.1183/23120541.00161-2023.Supp1 **Please note:** supplementary material is not edited by the Editorial Office, and is uploaded as it has been supplied by the author.
Figure S1 00161-2023.SUPPLEMENT
Figure S2 00161-2023.SUPPLEMENT2
Figure S3 00161-2023.SUPPLEMENT3
Figure S4 00161-2023.SUPPLEMENT4
