# Supplementary figures and images for: Identification of diurnal rhythmic blood markers in bronchial asthma
Source: ERJ Open Res. 2023 Jul 3;9(4):00161-2023. doi: 10.1183/23120541.00161-2023 (PMC10316035; doi:10.1183/23120541.00161-2023)

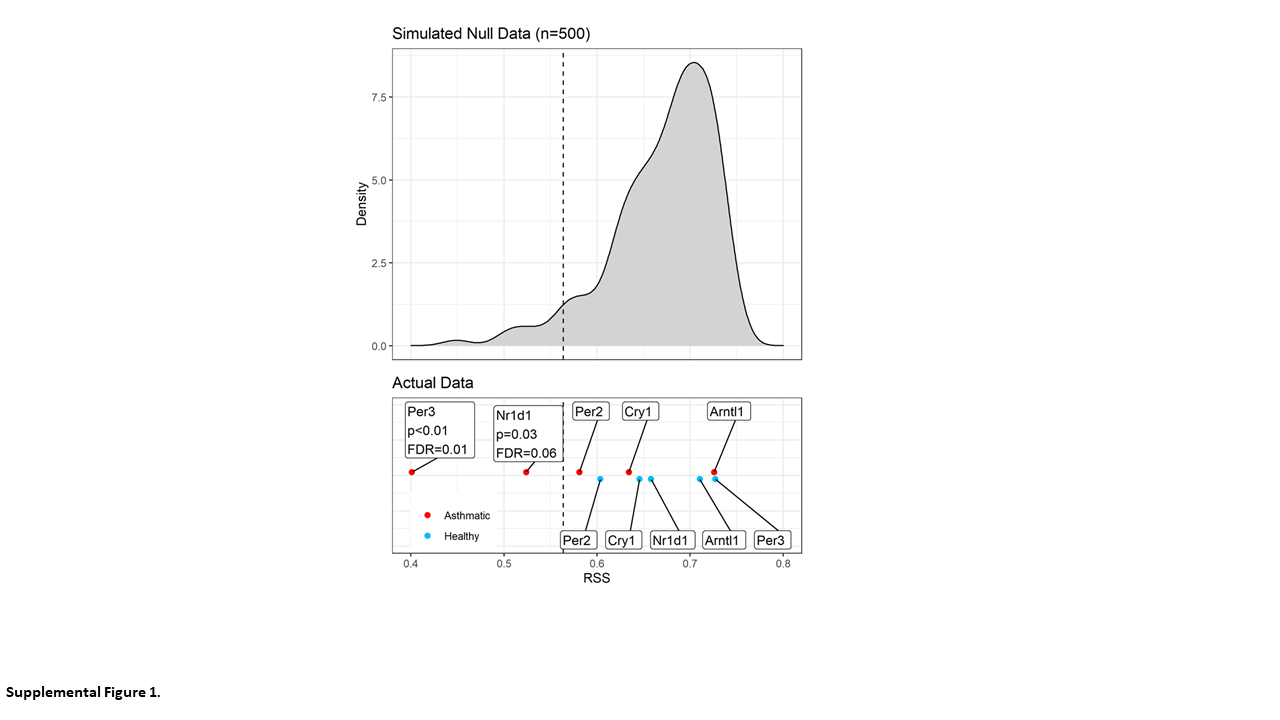

Supplement: Supplementary file 1 [file 00161-2023.SUPPLEMENT.tif]

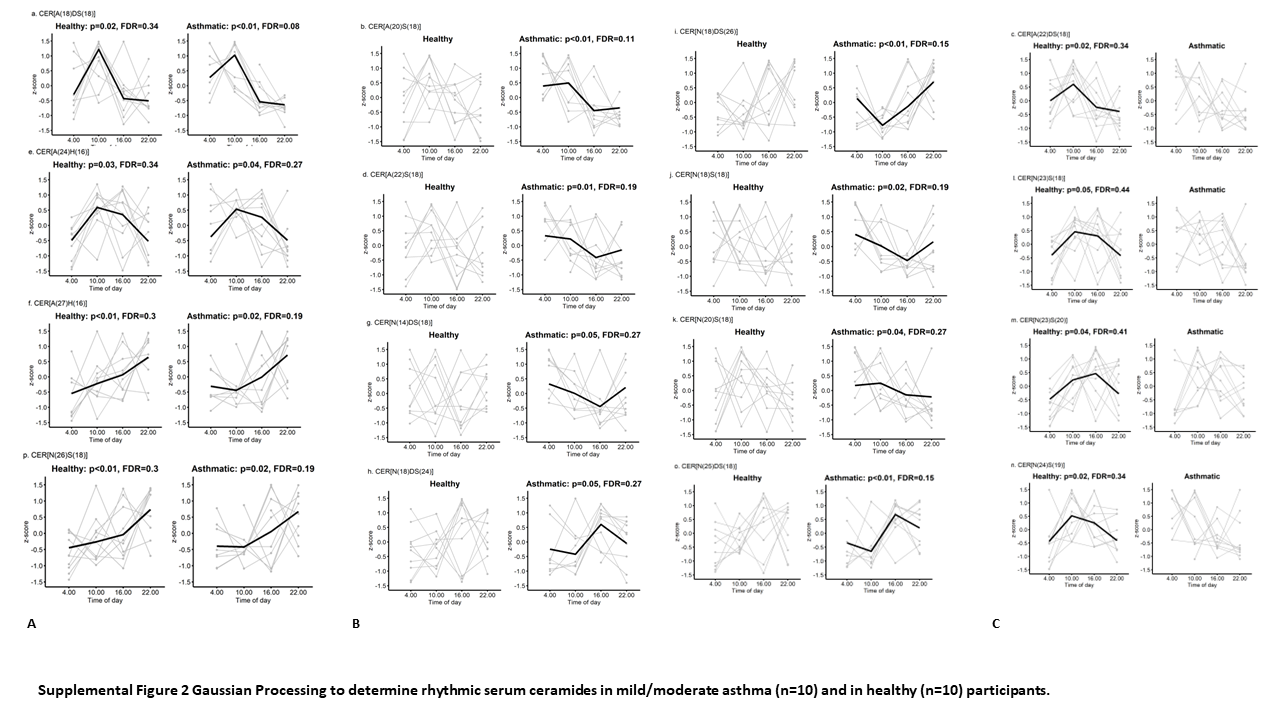

Supplement: Supplementary file 2 [file 00161-2023.SUPPLEMENT2.tif]

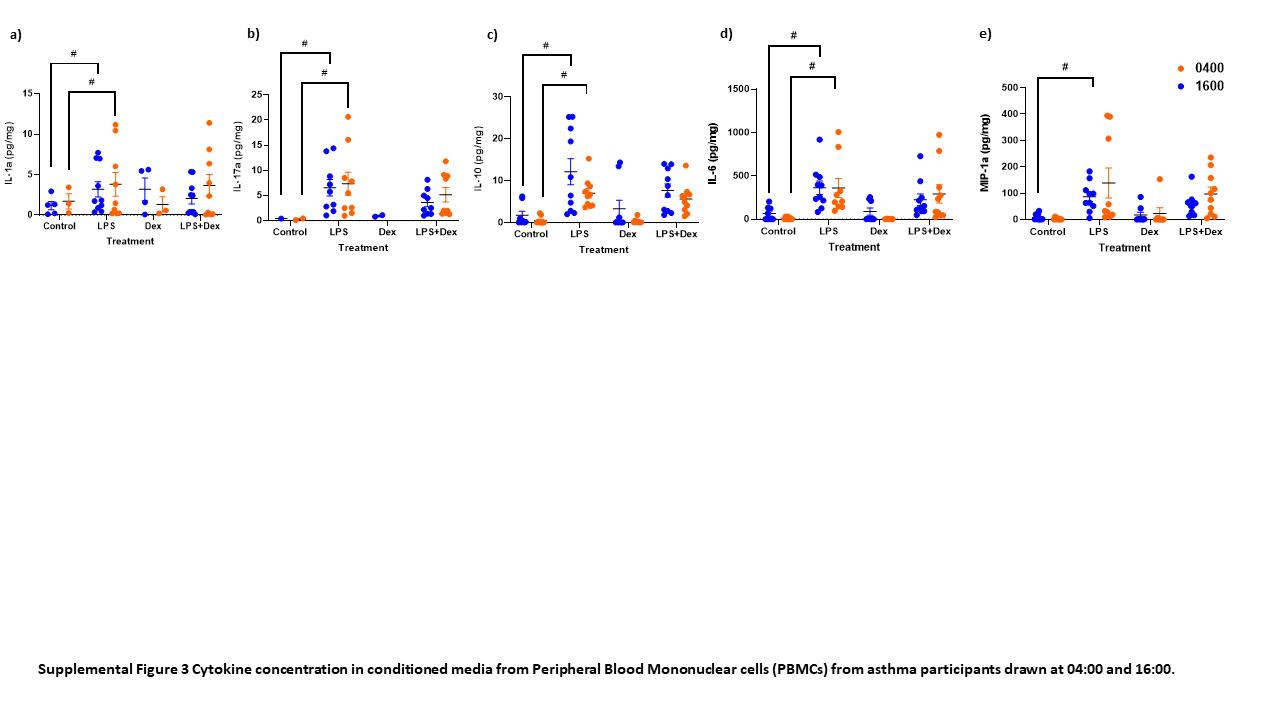

Supplement: Supplementary file 3 [file 00161-2023.SUPPLEMENT3.tif]

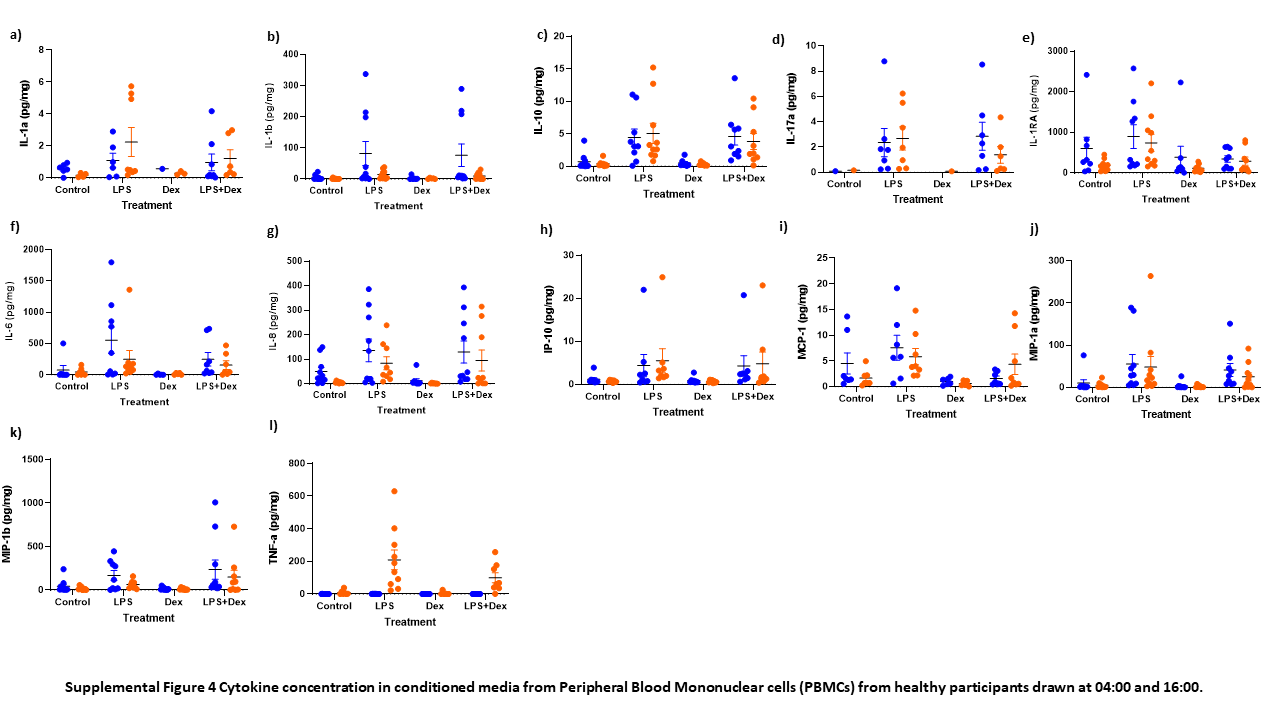

Supplement: Supplementary file 4 [file 00161-2023.SUPPLEMENT4.tif]
